# Effect of implant number on patient-reported outcomes in the edentulous maxilla: a systematic review and meta-analysis

**DOI:** 10.1186/s40729-026-00733-y

**Published:** 2026-10-27

**Authors:** Pascal Palmer, Stefan Wolfart, Jaana-Sophia Kern

**Affiliations:** Department of Prosthodontics and Biomaterials, Center for Implantology, Uniklinik RWTH Aachen, Pauwelsstr. 30, 52074 Aachen, Germany

**Keywords:** Dental implants, Edentulous maxilla, Oral health-related quality of life, Patient-reported outcome measures, OHIP, Meta-analysis, Implant number, Implant-supported prosthesis

## Abstract

**Purpose:**

This systematic review and meta-analysis evaluated changes in patient-reported outcomes following implant-supported rehabilitation of the edentulous maxilla and their association with implant number.

**Methods:**

Five databases were searched through August 2026. Randomized controlled and prospective clinical studies including ≥ 10 patients and reporting patient-reported outcomes before and after treatment were eligible. Retrospective studies, studies without baseline PROM assessment, and studies involving mandibular or simultaneous maxillary and mandibular implant treatment were excluded. Risk of bias was assessed using RoB 2, ROBINS-I, or MINORS, as appropriate. Hedges’ g was analyzed using multi-level random-effects meta-analysis, with sensitivity analyses for OHIP- and VAS-based outcomes.

**Results:**

Eleven studies were included. Patient-reported outcomes improved across all implant groups. Pooled effects were largest for ≥6 implants (g = 2.34; 95% CI: 1.85–2.83), followed by four implants (g = 1.13; 95% CI: 0.53–1.73); only one effect-size estimate was available for two implants. Improvement was significantly greater with ≥6 than with four implants (difference in g = 1.22; 95% CI: 0.40–2.04; *p* = 0.003) and remained significant when restricted to removable prostheses. Fixed and removable prostheses supported by six implants did not differ significantly. VAS-based outcomes showed a more pronounced difference than OHIP-based outcomes.

**Conclusions:**

Implant-supported rehabilitation substantially improves patient-reported outcomes in the edentulous maxilla. The greatest improvements were observed with ≥6 implants, with a significant advantage over four implants, although effect magnitude varied by PROM type. Evidence for two implants remains limited.

**Supplementary Information:**

The online version contains supplementary material available at https://doi.org/10.1186/s40729-026-00733-y.

## Introduction

Oral health is closely linked to general health and directly influences an individual’s quality of life [1]. Consequently, oral health-related quality of life (OHRQoL) has become an increasingly important focus in dental research as a key patient-reported outcome. Recognizing its importance, the World Dental Federation Assembly in 2015 emphasized that OHRQoL assessments should be integrated into oral health research to advance international health strategies [2].

Several validated instruments have been developed to measure dental patient-reported outcomes (dPROs), covering four major dimensions: oral function, psychosocial impact, orofacial pain, and orofacial appearance [3–5]. The Oral Health Impact Profile (OHIP) is the most widely used and extensively investigated instrument for assessing OHRQoL, while visual analog scales (VAS) are frequently used to assess specific patient-reported aspects of treatment [6]. The OHIP questionnaires exist in different variants including a long version (OHIP-49) and several short versions in different languages (e.g., OHIP-20, OHIP-19, OHIP-14, OHIP-5) [7].

Edentulism negatively affects OHRQoL across all domains. Beyond functional limitations, complete tooth loss also impacts speech, self-esteem, and esthetics. In an aging population, edentulism remains a public health challenge, although its prevalence varies substantially by geography and demographics [8, 9]. Implant-supported prostheses provide effective treatment for edentulous patients and generally improve OHRQoL compared with conventional complete dentures, particularly in the mandible [10, 11]. The baseline burden associated with conventional dentures differs substantially between the two arches. Mandibular complete dentures frequently provide limited retention and stability, resulting in functional impairment and dissatisfaction, whereas patients may adapt relatively well to conventional maxillary dentures because palatal coverage and an effective peripheral seal can provide favorable retention. Accordingly, implant treatment may produce particularly pronounced patient-reported improvements in the mandible. Consistent with these differences, implant overdentures have shown substantial improvements in patient satisfaction and OHRQoL compared with conventional complete dentures in the mandible, whereas patient-reported benefits in the maxilla appear more variable [12]. A systematic review reported that for individuals who are already comfortable with their complete maxillary dentures and have a well-preserved alveolar ridge, implant overdentures do not necessarily provide notable improvements in function, stability, or overall comfort [13]. Combined analyses of both arches may therefore overestimate treatment effects attributable to maxillary rehabilitation, as the larger improvements frequently observed after mandibular implant treatment may influence pooled estimates. Arch-specific analyses are therefore important when evaluating patient-reported outcomes following implant rehabilitation.

Treatment concepts for the edentulous maxilla vary considerably with respect to implant number and prosthetic design. Current recommendations support at least four implants for maxillary implant overdentures, while fixed full-arch rehabilitation may be performed using different implant numbers depending on the treatment concept and anatomical conditions [14–16]. This variability further complicates assessment of the independent contribution of implant number to patient-reported treatment benefit.

Recently, several systematic reviews originating from a global consensus initiative have comprehensively evaluated patient- and clinician-reported outcomes in the rehabilitation of the edentulous maxilla across different treatment concepts, including implant-retained overdentures, fixed prostheses, implant number, timing of placement and loading, and adjunctive procedures such as sinus floor elevation [17–23]. These efforts reflect the growing recognition of the importance of patient-reported outcomes in the evaluation of implant therapy and provide a structured overview of the current evidence base. These reviews report generally favorable patient-reported outcomes following implant therapy but also highlight considerable heterogeneity in study designs, treatment protocols, and outcome measures. However, the available evidence has largely been synthesized descriptively or within specific treatment concepts, and some analyses have included studies reporting treatment of both arches. Earlier systematic reviews have faced similar limitations, either combining maxillary and mandibular outcomes or lacking quantitative analyses restricted to the edentulous maxilla [12, 24–26].

Against this background, a need remains for analyses that quantitatively integrate available data and focus specifically on longitudinal changes in patient-reported outcomes in the edentulous maxilla.

The present systematic review and meta-analysis therefore aimed to quantify changes in patient-reported outcomes from before to after implant-supported rehabilitation and to investigate whether the magnitude of patient-reported improvement differs according to implant number. Fixed and removable prosthetic concepts were included, with additional analyses exploring the potential influence of prosthetic design and PROM type.

The PICO question was: *In patients with completely edentulous maxillae (P), how do patient-reported outcomes (O) change after treatment with implant-supported fixed or removable prostheses (I), compared with the pre-treatment condition and between different numbers of implants (C)?*

## Methods

### Protocol, registration, and structure

Prior to conducting the systematic literature search, a review protocol was established in accordance with the PROSPERO guidelines. The study was prospectively registered on PROSPERO international database for systematic reviews (registration number: CRD420251055557) available under: https://www.crd.york.ac.uk/PROSPERO/view/CRD420251055557

This systematic review and meta-analysis followed the PRISMA (Preferred Reporting Items for Systematic reviews and Meta-Analyses) guidelines [27].

### Eligibility criteria

The eligibility criteria were defined according to the PICO (Population, Intervention, Comparison, Outcome) scheme according to Richardson et al., 1995 and Sackett et al., 1996.

#### Inclusion criteria

- Population: Patients with completely edentulous maxillae.
- Intervention: Treatment with either a fixed or removable implant-supported prosthesis.
- Outcome: Patient-reported outcomes related to OHRQoL, patient satisfaction, or treatment-related domains, assessed using OHIP or VAS-based measures, with results reported after a minimum observation period of 6 months.
- Study design: Prospective clinical studies or randomized-controlled trials, including at least 10 patients per study/cohort.
- Language: Articles published in English.

#### Exclusion criteria

- **Population-related:**
  a Patients younger than 18 years of age.
  b Patients with edentulism due to syndromic diseases.
  c Patients treated with mini implants, zygoma implants, or any other special implant forms.
  d Patients receiving implant treatment exclusively in the mandible or simultaneously in mandible and maxilla.
- **Intervention/Outcome-related:**
  a Studies lacking baseline assessment of dPROMS prior to implant placement.
  b Studies with insufficient documentation of dPROMS.
- **Study design-related:**
  a Case reports.
  b Retrospective studies.

### Search strategy

A search strategy was developed following the PICO-scheme incorporating different spelling variations to maximize the number of identified studies, with modifications made to align with the unique characteristics of each database.

The search strategy combined Medical Subject Headings (MeSH) and free-text terms related to edentulous maxilla, dental implants, implant-supported prostheses, and oral health-related quality of life. The search terms included combinations of the following: #1(“edentulous Mouth“OR “Jaw, Edentulous” OR “Edentulous“) AND (“Maxilla“OR “Maxillary“) AND #2(“dental implants” OR “Dental Prosthesis” OR”implant-retained“OR “implant-supported” OR “denture*” OR “Overdenture*“OR “Complete Denture*“OR”Overlay”) AND #3(“Quality of Life” OR “Patient Satisfaction” OR “Satisfaction” OR “Patient* centered outcome*” OR “patient* outcome*” OR “oral health-related quality of life” OR “patient* related outcome*”)

Furthermore, a filter was implemented for the English language and for clinical trials. The search string was adapted according to the database in question. To ensure completeness of the search, the references of the included studies, related systematic reviews and additional suggestions from PubMed were examined manually.

### Study selection process

The electronic databases were searched independently by two reviewers (P.P. and J.-S.K.). In cases of disagreement, a third reviewer (S.W.) was consulted. The following databases were searched:

- PubMed Central (PMC): 1999–May, 18th 2025
- MEDLINE (via PubMed): 1987–May, 18th 2025
- Embase: 1947–May, 18th 2025
- Scopus: 1971–May, 18th 2025
- CENTRAL (Cochrane Library): 1950–May, 18th 2025

**Fig. 1:**
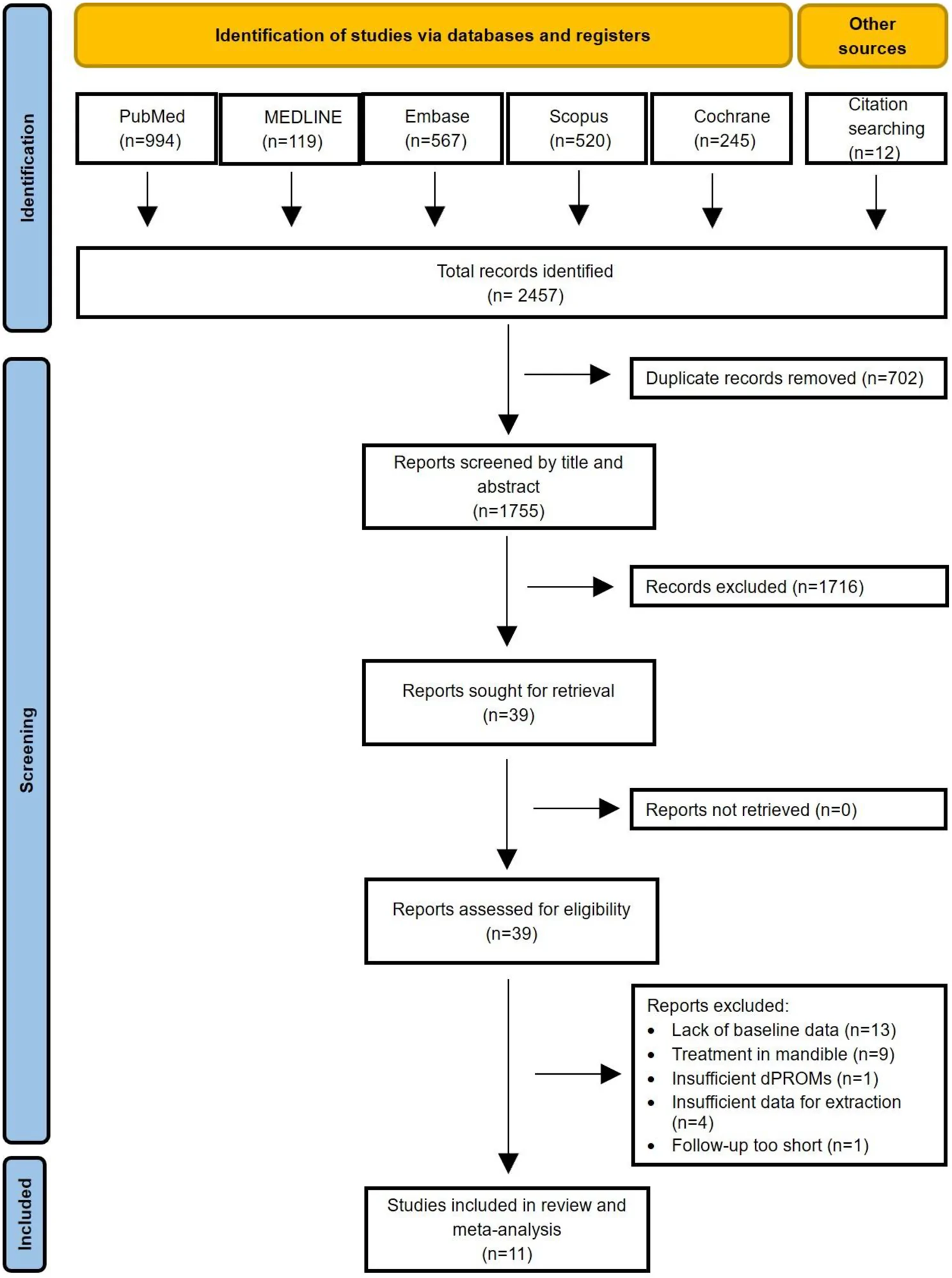
PRISMA flow diagram of the study selection process

The initial electronic search was conducted on May 18, 2025, and the search was updated in August 2026 using the same search strategy. After duplicate removal using Rayyan.ai (https://www.rayyan.ai/), all remaining records were screened by title and abstract to identify potentially relevant studies. Consensus on full-text eligibility was reached, and selected articles were managed in EndNote (version 21, Clarivate, London, UK). Final screening was performed according to the predefined inclusion and exclusion criteria. The study selection process is illustrated in the PRISMA flow diagram (Fig. 1).

Data were extracted independently by two reviewers (P.P. and J.S.K.) using a standardized Excel sheet. Extracted information included publication details, study design, participant characteristics, number of implants, type of prosthesis, follow-up duration, and outcomes related to oral health-related quality of life (e.g., OHIP scores, VAS scores). In case of disagreement, a third reviewer (S.W.) was consulted. Inter-reviewer agreement was high at both the title/abstract and full-text screening stages, with perfect agreement for full-text inclusion (Cohen’s κ = 1.00).

For the meta-analysis, mean values, standard deviations (SD), and sample sizes were extracted. All reported dPROMS, including different versions of the Oral Health Impact Profile (OHIP-14, OHIP-20, OHIP-49) and visual analogue scales (VAS), were systematically collected. Follow-up intervals were recorded as reported. Decisions regarding which outcome measures and follow-up points could be meaningfully compared and statistically pooled were made during the meta-analytic phase, based on conceptual and methodological comparability.

When only mean values and 95% confidence intervals (95% CI) were available, SDs were calculated accordingly. If only medians with interquartile ranges or minimum and maximum values were reported, means and SDs were estimated based on established methods [28]. Different OHIP versions (e.g., OHIP-14, OHIP-20, OHIP-49) and VAS measures were transformed into standardized effect sizes (Hedges’ g), allowing comparison across instruments with different scoring ranges. In cases of missing data, study authors were contacted.

### Risk of bias assessment

The risk of bias and methodological quality of the included studies were independently assessed by two reviewers (P.P. and J.S.K.). Disagreements were resolved through discussion and, if necessary, by consultation with a third reviewer (S.W.). Randomized controlled trials were assessed using the revised Cochrane Risk of Bias tool (RoB 2), non-randomized comparative studies using the Risk Of Bias In Non-randomized Studies of Interventions (ROBINS-I) tool, and non-randomized single-arm studies using the Methodological Index for Non-Randomized Studies (MINORS).

### Statistical analysis

Data from individual studies were included in the meta-analyses only if the mean and standard deviation (SD) were either explicitly reported or could be estimated from the median and interquartile range using established methods [28]. For each outcome variable, effect sizes (Hedges’ *g*) were calculated from baseline and follow-up data, following the methodology described by Goulet-Pelletier and Cousineau [29]. When required, means and standard deviations were estimated from median and interquartile range, as indicated in the dataset.

Effect sizes were calculated comparing two follow-up periods (12 to 24 months, and 36 to 60 months) to baseline (month 0). Positive effect sizes indicate an improvement in the respective outcome, whereas negative values indicate deterioration. As the correlation between baseline and follow-up measurements was not reported in the included studies, and because sample sizes differed between time points in some cases, Hedges’ *g* was calculated under the assumption of independent samples. To account for potential dependencies of multiple effect sizes derived from the same study (e.g., multiple PROMs or subgroups) and to reduce the risk of pseudo-replication of patient data, a multi-level random-effects meta-analysis model was applied. This approach allowed the inclusion of multiple outcome measures from the same study while accounting for their statistical dependence. Consequently, studies reporting numerous PROM domains did not contribute the same amount of independent information as would be assumed in a conventional two-level meta-analysis, thereby reducing their potential influence on the pooled estimates. Thus, the number of observations included in the meta-analysis may exceed the number of unique patients, as multiple effect-size estimates could originate from the same study population. Overall effect sizes with corresponding 95% confidence intervals (95% CI) were estimated using restricted maximum likelihood (REML).

To account for potential differences in measurement properties between outcome instruments, separate analyses were performed for OHIP-based and VAS-based outcomes. While the OHIP represents a validated multidimensional instrument for assessing oral health-related quality of life, VAS measures reflect simpler, often single-domain assessments. These sensitivity analyses were conducted to evaluate whether the observed treatment effects were consistent across different types of patient-reported outcome measures and to avoid potential bias arising from pooling fundamentally different instruments.

Statistical heterogeneity in the primary meta-analyses was assessed using Cochran’s Q statistic and corresponding *p*-values. Subgroup analyses were performed using random-effects meta-regression. Forest plots were generated to graphically illustrate the effect size estimates.

Potential publication bias was explored by generating funnel plots for the main analyses. Given the limited number of independent studies and the inclusion of multiple correlated effect-size estimates within several studies, funnel plots were considered exploratory and interpreted with caution.

All statistical tests were two-sided with a significance level set at 0.05. Statistical analyses were conducted in R (R Foundation for Statistical Computing, Vienna, Austria).

## Results

### Selection of studies

The systematic literature search, including both database and manual searches, yielded 2,457 records. After removal of duplicates, 1,755 records remained for title and abstract screening. Of these, 39 studies were considered potentially eligible and were assessed in full text.

The most frequent reasons for exclusion at full-text assessment were the absence of baseline PROM assessment (*n* = 13) and simultaneous implant treatment in the mandible (*n* = 9). The updated literature search did not lead to further inclusion of full-texts.

Ultimately, 11 studies [30–40] met the inclusion criteria and were included in the qualitative and quantitative analyses (see Table 1). Fig. 1 shows all details of the study selection process in a PRISMA flow chart. Where studies reported data from the same cohort at different follow-up periods, each cohort was included only once per follow-up interval in the meta-analysis to avoid duplicate inclusion of the same data.

**Table 1:** Study characteristics

Author (year)	Study design/type of prosthesis/type of attachment	Follow-up (months)	Number of patients (baseline/follow-ups)	Implants per patient per group	PROMs	Outcome: Baseline and follow-ups	
						Mean/Standard Deviation	Median/Q25/Q75
Bernard et al. (2019)	RCT (immediate vs. conventional loading)/fixed/cone anchored screw-retained	12/24	**Group 1:** Baseline: 7 Follow up: 7 **Group 2:** Baseline: 8 Follow-up: 8	**Group 1: 6** (immediate loading) **Group 2: 6** (conventional loading)	Total Satisfaction Score (calculated from 20 individual questions (VAS)	**Group 1:** 65.5 ± 14.9 84.5 ± 7.3 87.0 ± 5.4 **Group 2:** 63.7 ± 8.2 87.6 ± 2.9 91.0 ± 3.6	n.a.
Bouhy et al. (2023)	prospective/removable/Locator	12/36/60	Baseline: 30 Follow-ups: 29/27/27	**4**	OHIP-20	49.2 ± 17.3 28.7 ± 7.21 36.6 ± 17.9 39.0 ± 18.3	n.a.
Eerdekens et al. (2015)	prospective/removable/cone-anchored	12/36/60	Baseline: 10 Follow-ups: 10/10/10	**6**	Comfort, Function, Esthetics, Feeling as own teeth, Speech function, Self-confidence (VAS)	**Comfort:** 34.6 ± 21.5 82.6 ± 22.7 89.2 ± 10.4 89.5 ± 10.6 **Function:** 36.3 ± 28.8 92.2 ± 13.1 90.7 ± 12.2 92 ± 12.2 **Esthetics:** 58.7 ± 36.6 93.7 ± 10.2 89.8 ± 10 85.9 ± 14.1 **Feeling as own teeth:** 25.7 ± 22.5 83.7 ± 17.7 84.9 ± 17.4 86.9 ± 12 **Speech function:** 56.6 ± 30.9 94.4 ± 6.2 93 ± 8.2 93 ± 8.2 **Self-confidence**: 38.2 ± 25.6 81.9 ± 25.5 90.4 ± 10.7 90.4 ± 10.7	n.a.
Onclin et al. (a2023)	RCT (Locator vs. bar)/ Removable/Locator or bar	12/60	**Group 1:** Baseline: 25 Follow-ups: 25/25 **Group 2:** Baseline: 25 Follow ups: 25/25	**Group 1: 4** (locator) **Group 2: 4** (bar)	OHIP-49 General Satisfaction Score (VAS)	n.a.	**Group 1:** 77/59.5/120.5 26/9/61 21/8/46 **Group 2:** 84/52/105.5 12.5/3/36.5 8/2/43.5
Onclin et al. (b2023)	RCT (2 vs. 4 implants)/ removable/bar	12	**Group 1:** Baseline: 18 Follow-up: 18 **Group 2:** Baseline: 18 Follow-up: 18	**Group 1: 2** **Group 2: 4**	OHIP-49 Chewing Ability Questionnaire	n.a.	**Group 1:** 63.5/31.8/92.8 16/12/20 **Group 2:** 69.5/31.3/83 17/8/27
Erkapers et al. (2017)	Prospective/fixed/screw-retained full-arch bridge	3/6/12/24/36	Baseline: 51 Follow-ups: 47/46/47/46/45	**6**	OHIP-49	107.0 ± 37.2 78.2 ± 33.3 70,4±23.5 63.5 ± 17.2 63.7 ± 20 63.4 ± 17.8	n.a.
Markovic et al. (2024)	RCT/fixed/screw-retained full-arch bridge	24	**Group 1:** 12/12/12 **Group 2:** 12/12/12	**Group 1: 6** (immediate loading) **Group 2: 6** (early loading)	OHIP-19	**Group 1:** 21.6 ± 8.5 2.2 ± 1.5 1.5 ± 1.7 **Group 2:** 17.9 ± 8.8 2.1 ± 2,2 1.8 ± 2.8	n.a.
Zembic et al. (2019)	prospective/removable/Locator	12/48	Baseline: 21 Follow-up: 16	**2**	OHIP-20 “functional limitation”	n.a.	n.a.
Fonteyne et al. (2021)	prospective/removable/bar	36	Baseline: 21 Follow-up: 21	**4**	OHIP-14 General Satisfaction Score (VAS)	**OHIP:** 64.5 ± 26.6 81.7 ± 13.3 **VAS:** 21.5 ± 12.2 6.1 ± 4.9	n.a.
Peñarrocha-Oltra et al. (2014)	prospective/fixed/screw-retained full-arch bridge	3/12	**Group 1:** Baseline: 15 Follow-up: 14/14 **Group 2:** Baseline: 15 Follow-up: 15/15	**Group 1: 6–8** (immediate loading) **Group 2: 6–8** (conventional loading)	General Satisfaction Score (VAS)	**Group 1:** 45 ± 18 83 ± 11 89 ± 7 **Group 2:** 48 ± 17 49 ± 13 88 ± 10	n.a.
Park et al. (2019)	RCT (bar vs. ball attachment)/removable/bar or ball	3/12	**Group 1:** Baseline: 20 Follow-up: 20/20 **Group 2:** Baseline: 20 Follow-up: 20/20	**Group 1: 4** (bar) **Group 2: 4** (ball)	General Satisfaction Score (VAS)	**Group 1:** 91 ± 11 89 ± 18 93 ± 16 **Group 2:** 94 ± 10 94 ± 11 94 ± 10	n.a.

### Risk of bias across included studies

The risk of bias was assessed separately for randomized controlled trials (RCTs), non-randomized comparative studies, and non-randomized single-arm studies.

Among the RCTs assessed with RoB 2, most studies were judged as having either low risk of bias or some concerns. Randomization and allocation procedures were generally adequate. The main limitation was the measurement of outcomes, as blinding of participants and outcome assessors was rarely feasible for subjective PROM-based assessments. Selective reporting was uncommon, as most trials reported prespecified outcomes (Table 2).

**Table 2:**
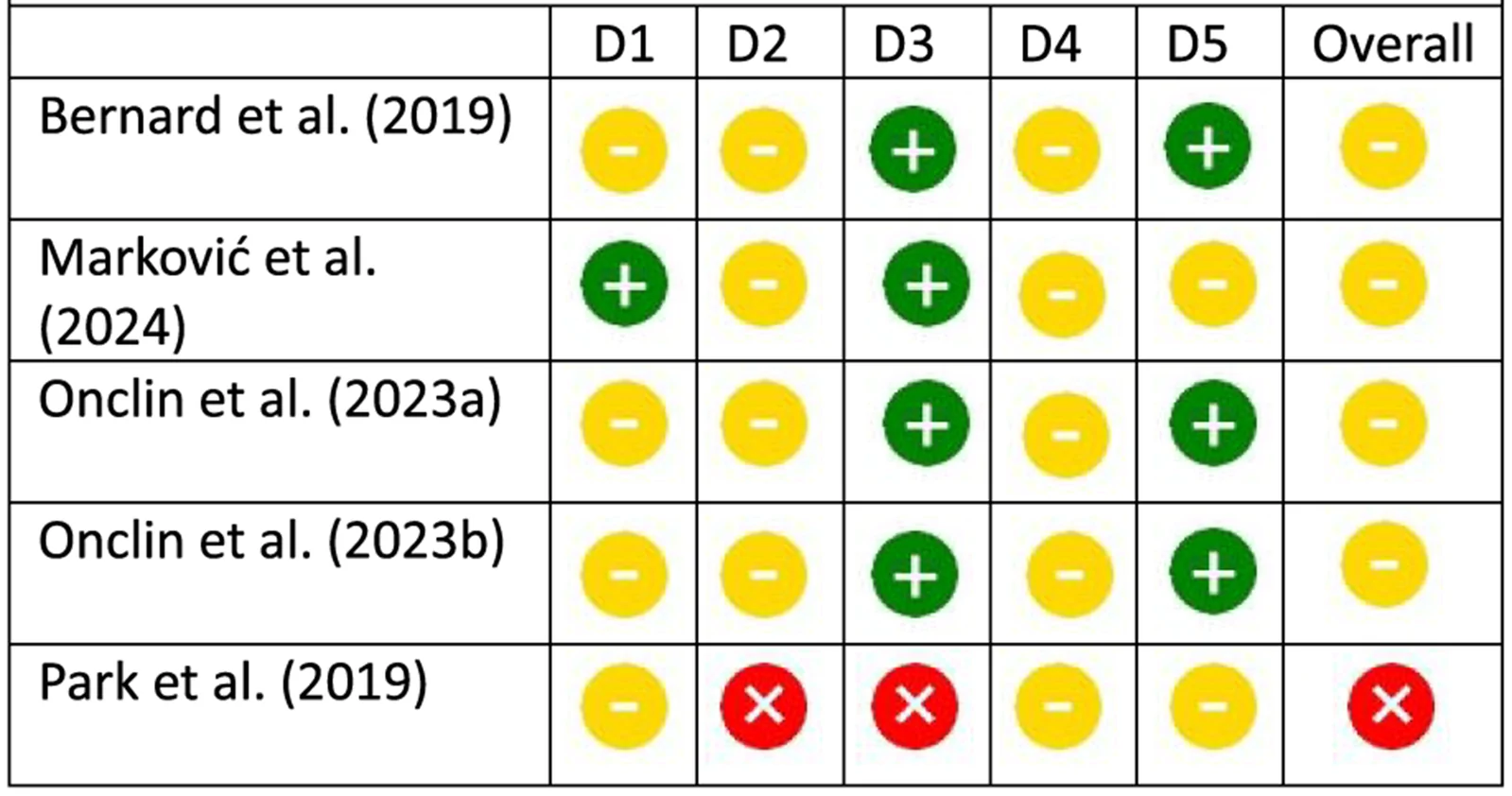
Risk of bias analysis of included RCTs (RoB 2)

Non-randomized comparative studies assessed with ROBINS-I were predominantly rated as having moderate risk of bias. The main sources of bias were confounding and participant selection, as treatment allocation often depended on clinical indication or patient preference without adjustment for confounders. Outcome measurement was commonly unblinded and based on subjective PROMs. Selective reporting was not a major concern (Table 3).

**Table 3:**
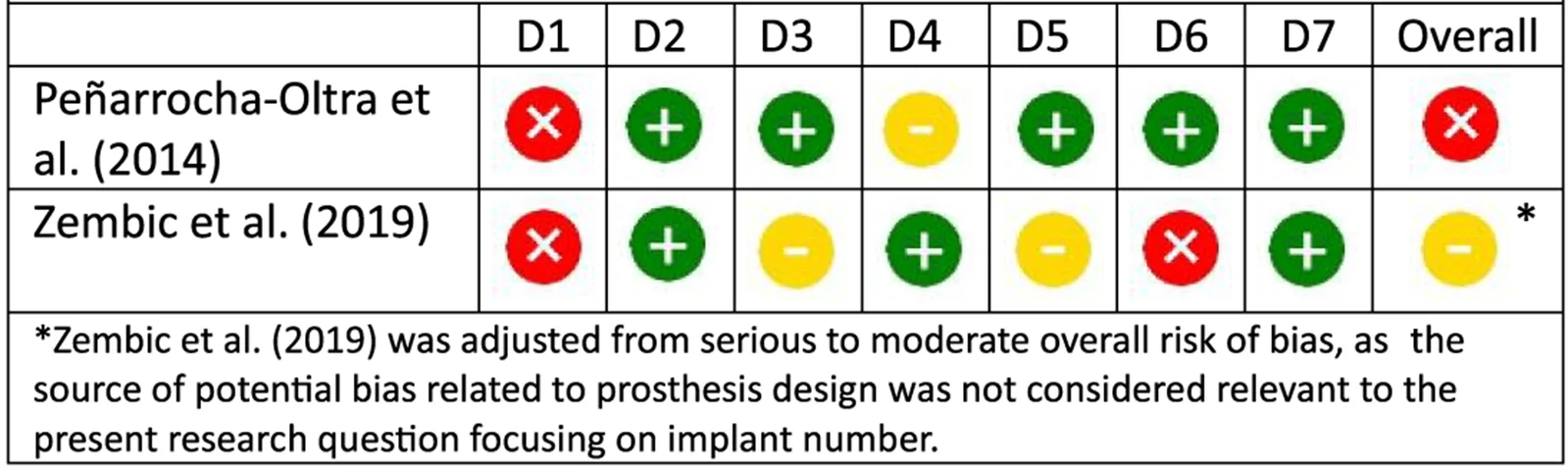
Risk of bias analysis of included non-randomized comparative studies (ROBINS-I)

Non-randomized single-arm studies assessed with MINORS scored between 12 and 14 out of 16 points. These studies generally stated their aims clearly, included consecutive patients, and collected data prospectively. The main limitations were insufficient reporting on unbiased outcome assessment, incomplete documentation of loss to follow-up, and lack of prospective sample size calculation (Table 4).

**Table 4:**
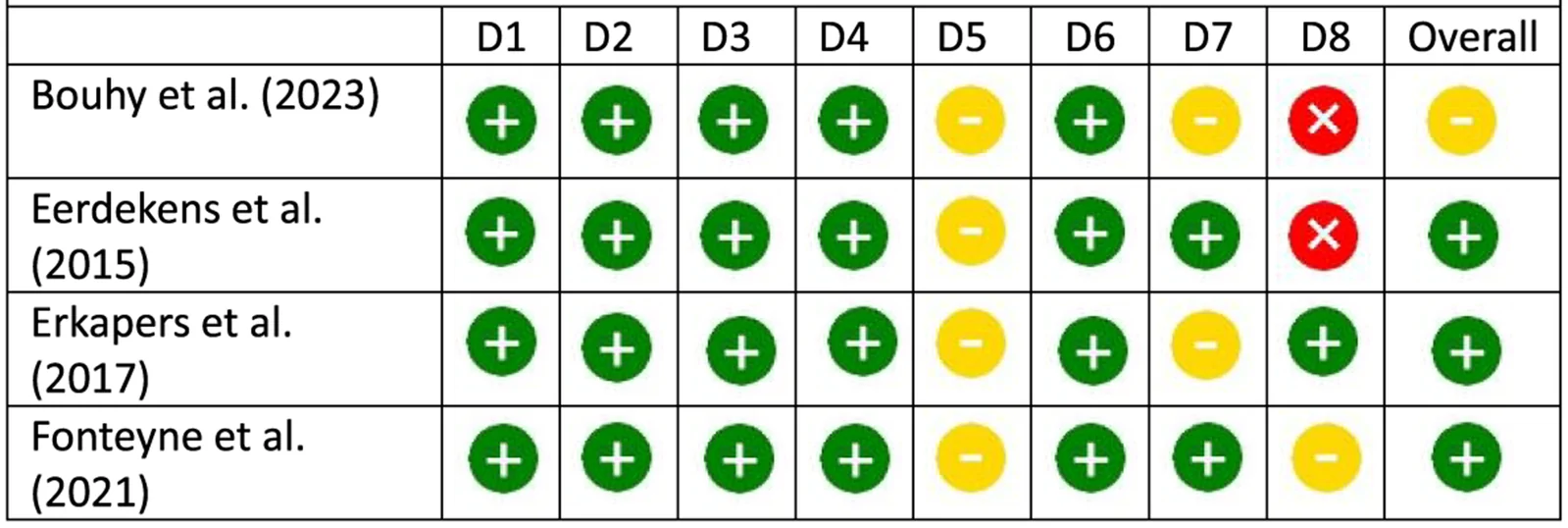
Risk of bias analysis of included non-randomized single-arm studies (MINORS)

Overall, the included evidence was judged to have a low to moderate risk of bias for randomized studies, predominantly moderate risk of bias for non-randomized comparative studies, and acceptable methodological quality for single-arm studies. Detailed domain-level assessments are provided in Tables 2, 3, 4.

### Measures and follow-up periods

The included studies applied different dPROMS and follow-up periods. To improve comparability across studies, the selection of dPROMS and timing of outcome assessment were standardized for the meta-analysis. All included studies used either a version of the Oral Health Impact Profile (OHIP) or visual analogue scales (VAS). For studies reporting OHIP outcomes, the total OHIP score was used whenever available rather than individual subdomains. As no equivalent overall score exists for VAS-based assessments, individual VAS outcomes were included as separate effect-size estimates. Standardization as Hedges’ *g* allowed outcomes measured on different scales to be combined in the primary analysis. To examine whether the results were influenced by the type of outcome instrument, sensitivity analyses were additionally performed separately for OHIP- and VAS-based outcomes. An overview of the PROMs used in the included studies is provided in Table 5.

**Table 5:** Follow-up periods of the included studies according to implant number

	Author (year)	Type of prosthesis	Follow-up [months]							
			1	3	6	12	24	36	48	60
2 implants	Onclin et al. (2023)	removable	-	-	-	x	-	-	-	-
	Zembic et al. (2019)	removable	-	-	-	x	-	-	x	-
4 implants	Onclin et al. (2023)	removable	-	-	-	x	-	-	-	x
	Park et al. (2019)	removable	-	x	-	x	-	-	-	-
	Fonteyne et al. (2021)	removable	-	-	-	-	-	x	-	-
	Bouhy et al. (2023)	removable	-	-	-	x	-	x	-	x
≥6 implants	Eerdekens et al. (2015)	removable	-	-	-	x	-	x	-	x
	Erkapers et al. (2017)	fixed	-	x	x	x	x	x	-	-
	Bernard et al. (2019)	fixed	-	-	-	x	x	-	-	-
	Peñarrocha-Oltra et al. (2014)	fixed	-	x	-	x	-	-	-	-
	Markovic et al. (2024)	fixed	x	-	-	-	x	-	-	-

For the primary meta-analysis, outcome assessment was restricted to follow-up periods between 12 and 24 months, as this interval provided the greatest comparability across studies. Longer follow-up periods were not combined with the primary analysis because treatment effects may change over time. Where sufficient data were available, later follow-up periods (36−60 months) were considered separately in subgroup analyses (Table 5).

### Main meta-analysis (12–24 months)

Ten studies contributed data to the primary meta-analysis. Studies were not included in the primary analysis if no overall OHIP score was available or if outcomes were reported only at a follow-up time point outside the predefined 12 − 24-month interval. Where appropriate, these studies were considered in subsequent subgroup or sensitivity analyses. For the primary analysis, studies were stratified according to implant number into three groups: 2, 4, and ≥6 implants. The category “≥6 implants” was used because the number of implants within this group ranged from six to eight.

As some studies reported multiple VAS outcomes from the same patient cohort, multiple effect-size estimates could originate from a single study population. To account for the resulting statistical dependence and avoid pseudo-replication of patient data, a multi-level random-effects meta-analysis was applied. This approach allowed multiple outcomes from the same study to be included while accounting for their within-study clustering.

#### Two implants

For the two-implant group, only one study by Onclin et al. [31], including 18 patients, contributed to the primary analysis. A large improvement in OHRQoL was observed at follow-up (Hedges’ *g* = 1.30; 95% CI: 0.55 − 2.05). Zembic et al. could not be included in the primary analysis because no overall OHIP-20 score was reported. As only one effect-size estimate was available, a pooled multi-level estimate could not be calculated for the two-implant group.

#### Four implants

**Fig. 2:**
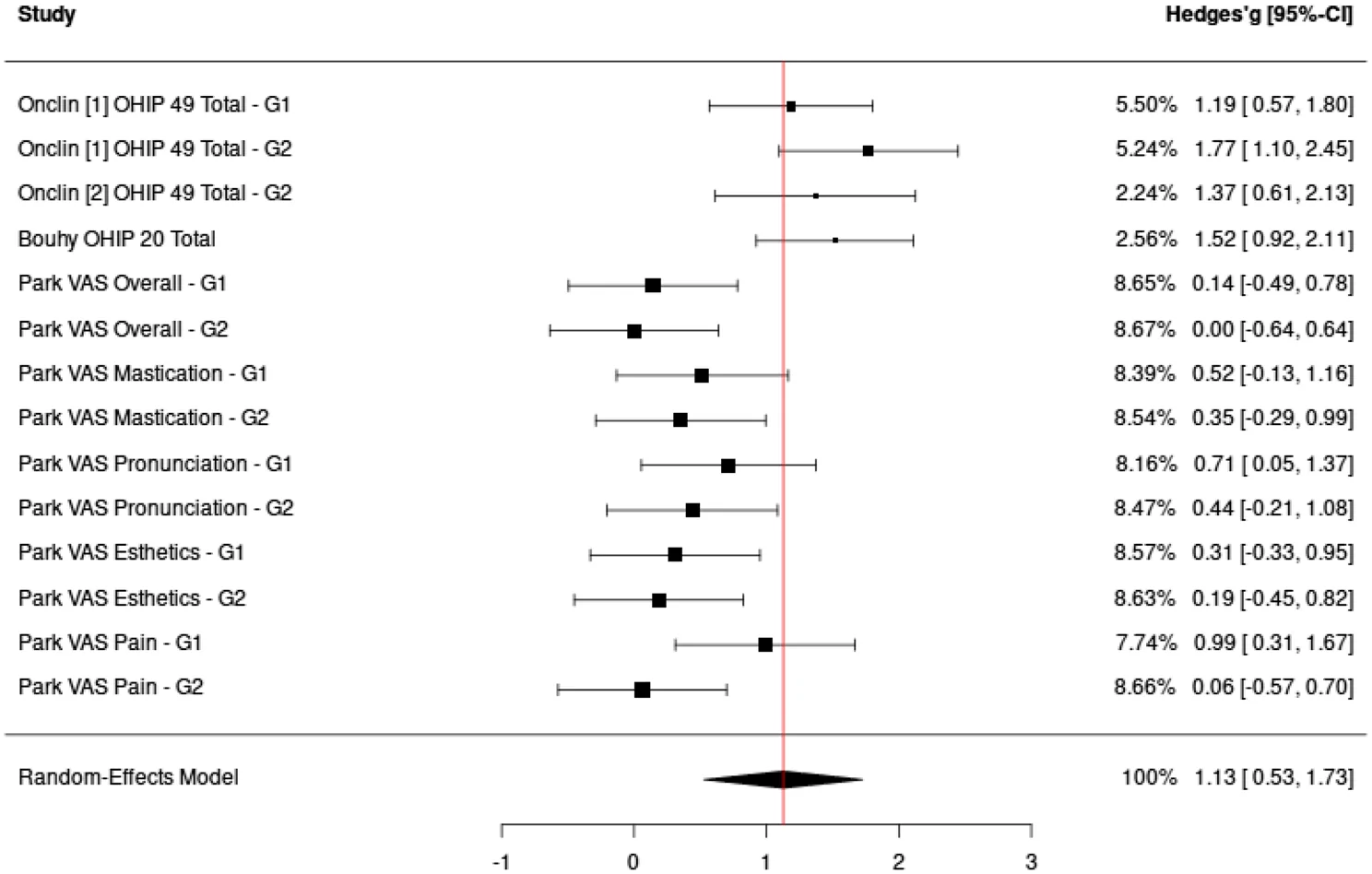
Forest plot of dPROMS for four-implant removable prostheses at 12–24 months. Effect sizes are expressed as Hedges’ g with 95% confidence intervals

**Fig. 3:**
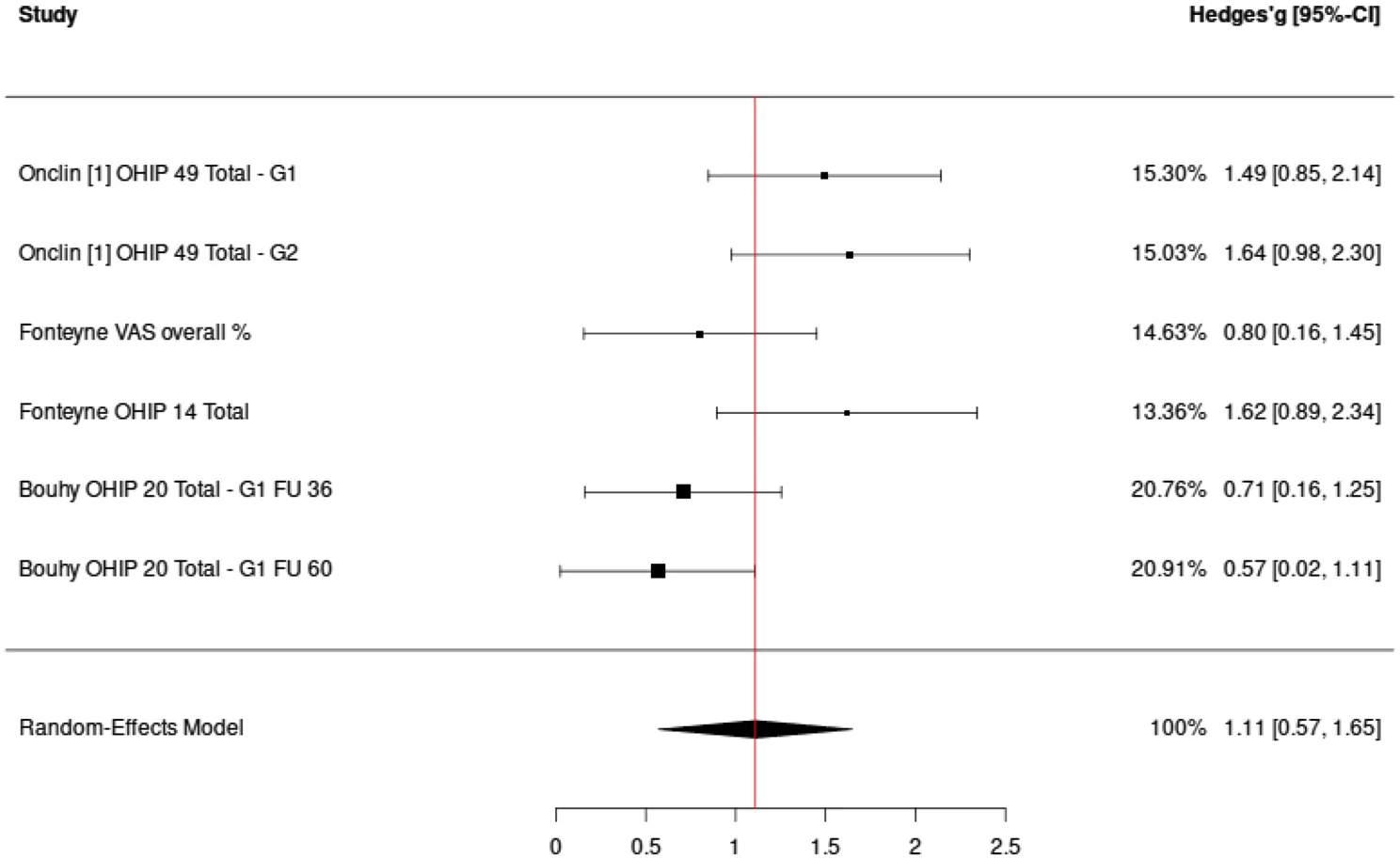
Forest plot of dPROMS for four-implant removable prostheses at 36–60 months. Effect sizes are expressed as Hedges’ g with 95% confidence intervals

For the four-implant group, 14 effect-size estimates were included in the multi-level meta-analysis (Fig. 2). All patients were rehabilitated with removable prostheses. The pooled effect size indicated a large improvement in patient-reported outcomes (Hedges’ *g* = 1.13; 95% CI: 0.53 − 1.73). Significant heterogeneity was observed across effect-size estimates (Q = 40.3; *p* < 0.001). At longer-term follow-up (36–60 months), the improvement remained of similar magnitude (Hedges’ *g* = 1.11; 95% CI: 0.57 − 1.65; Fig. 3).

#### Six or more implants

**Fig. 4:**
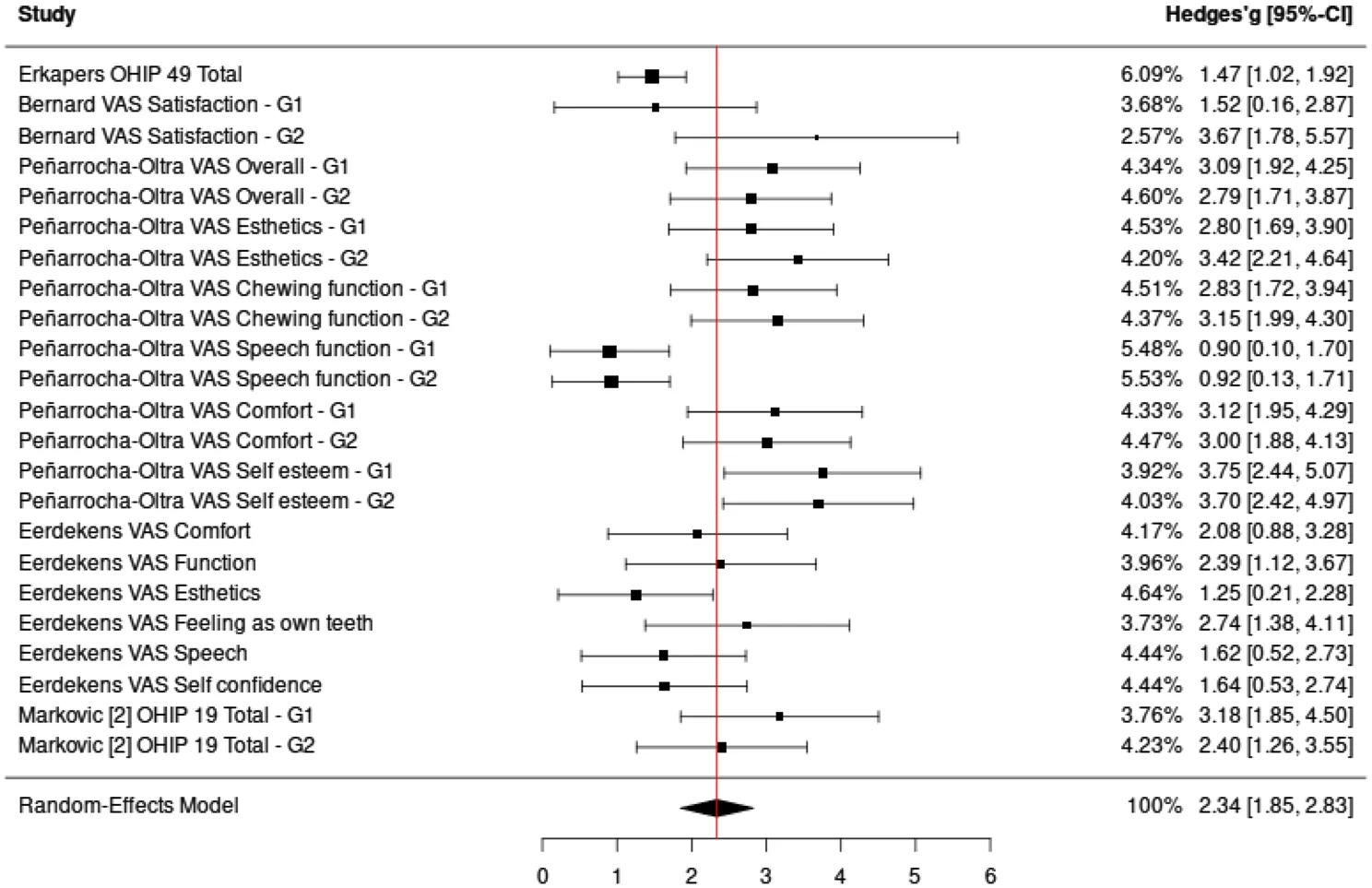
Forest plot of dPROMS for prostheses supported by six or more implants at 12–24 months. Fixed and removable prostheses were included. Effect sizes are expressed as Hedges’ g with 95% confidence intervals

**Fig. 5:**
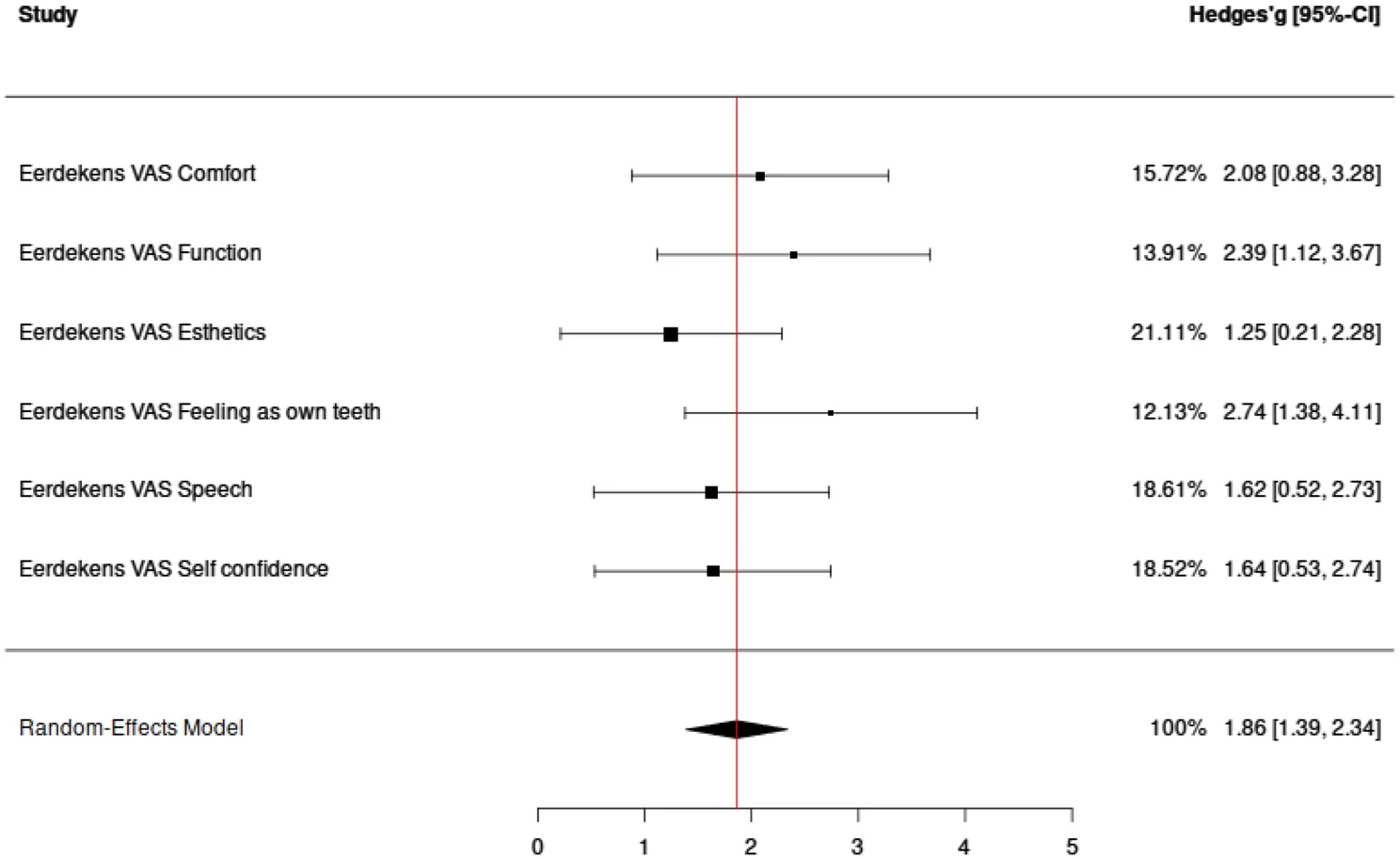
Forest plot of dPROMS for six-implant removable prostheses at 12–24 months. Effect sizes are expressed as Hedges’ g with 95% confidence intervals

**Fig. 6:**
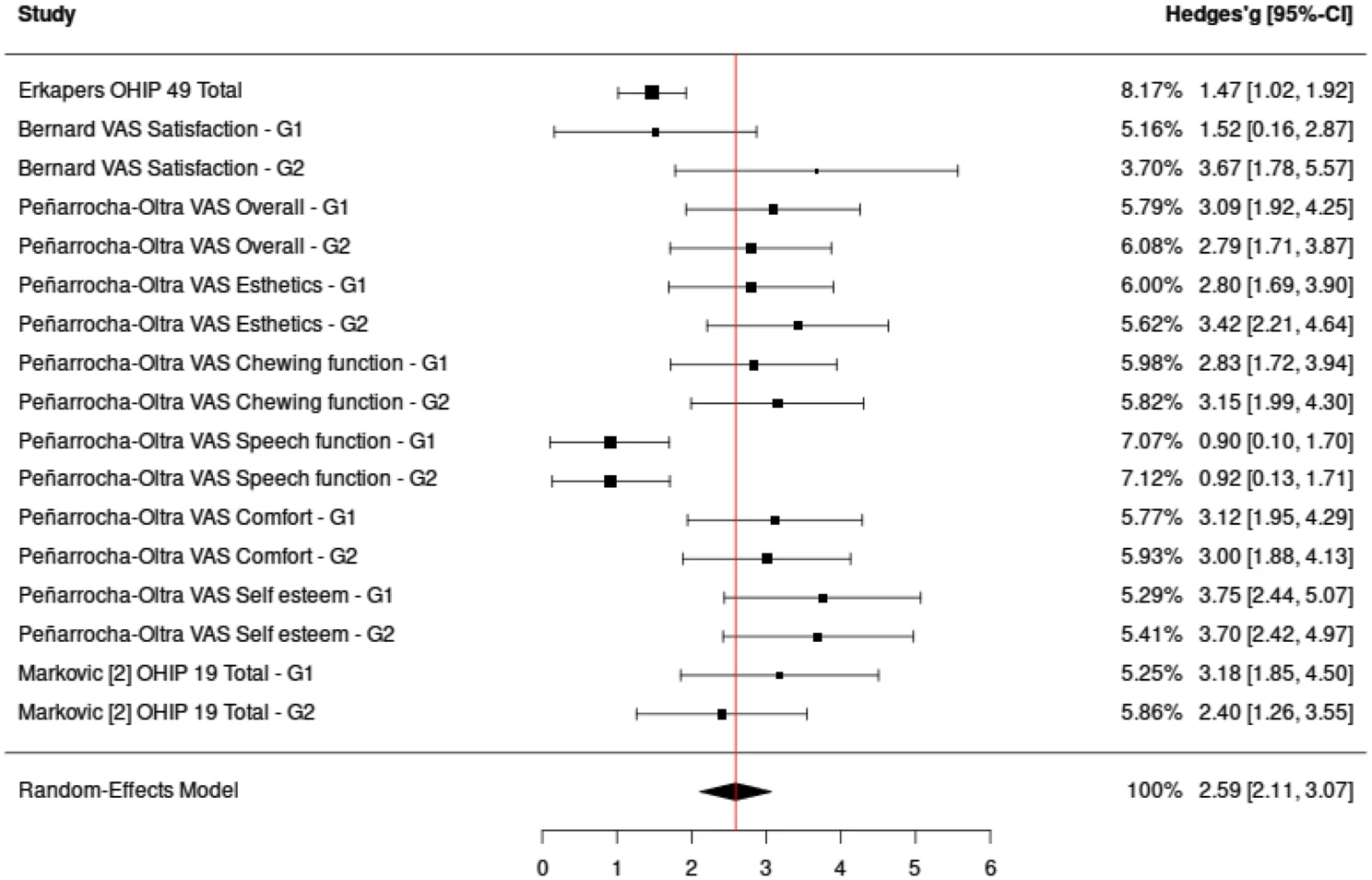
Forest plot of dPROMS for six-implant fixed prostheses at 12–24 months. Effect sizes are expressed as Hedges’ g with 95% confidence intervals

For the ≥6-implant group, 23 effect-size estimates were included in the multi-level meta-analysis at 12 − 24 months (Fig. 4). In contrast to the four-implant group, this group comprised both fixed and removable implant-supported prostheses. The pooled effect size indicated a large improvement in patient-reported outcomes (Hedges’ *g* = 2.34; 95% CI: 1.85 − 2.83). Significant heterogeneity was observed across effect-size estimates (Q = 68.6; *p* < 0.001). When the analysis was restricted to removable prostheses supported by six implants, the pooled effect size at 12 − 24 months was Hedges’ *g* = 1.86 (95% CI: 1.39 − 2.34; Fig. 5), whereas fixed prostheses supported by six implants showed a pooled effect size of *g* = 2.59 (95% CI: 2.11–3.07; Fig. 6).

### Comparison between implant groups

Pairwise comparisons using multi-level random-effects meta-regression showed a significantly greater improvement in patient-reported outcomes for rehabilitation with ≥6 implants compared with four implants (difference in Hedges’ *g* = 1.22; 95% CI: 0.40–2.04; *p* = 0.003). No statistically significant differences were observed between four and two implants (difference in Hedges’ *g* = −0.16; 95% CI: −1.62−1.29; *p* = 0.827) or between ≥ 6 and two implants (difference in Hedges’ *g* = 1.06; 95% CI: −0.46−2.58; *p* = 0.162) (Table 6).

**Table 6:** Comparison of dPROM effect sizes with different implant numbers irrespective of type of prosthesis

PROMS	n implants per patient	n effect size estimates	effect size [95%-CI]	p-value
OHIP total, VAS 12–24 months	2 implants 4 implants ≥6 implants 4 vs 2 (RE*) ≥6 vs 2 (RE*) ≥6 vs 4 (RE*)	1 14 23	− 1.13 [0.53, 1.73] 2.34 [1.85, 2.83] −0.16 [−1.62, 1.29] 1.06 [−0.46, 2.58] 1.22 [0.40, 2.04]	0.827 0.162 0.003

*RE: random-effects meta-regression

### Sensitivity analyses according to PROM type

To assess whether the results of the primary analysis were influenced by the type of outcome instrument, sensitivity analyses were performed separately for OHIP- and VAS-based outcomes.

#### OHIP-based outcomes

When the analysis was restricted to OHIP-based outcomes, improvements in OHRQoL were observed across all implant groups. For the two-implant group, only one effect-size estimate was available (Hedges’ *g* = 1.30; 95% CI: 0.55–2.05), precluding calculation of a pooled multi-level estimate. The pooled effect size was 1.46 (95% CI: 1.13–1.78) for four implants and 2.05 (95% CI: 0.81–3.28) for ≥6 implants.

Although the effect size was numerically larger for ≥6 implants, pairwise multi-level meta-regression showed no statistically significant differences between implant groups (four vs. two implants: difference in Hedges’ *g* = 0.12; 95% CI: −0.90 to 1.13; *p* = 0.819; ≥6 vs. two implants: 0.61; 95% CI: −0.58- 1.79; *p* = 0.316; ≥6 vs. four implants: 0.49; 95% CI: −0.36−1.34; *p* = 0.262).

#### VAS-based outcomes

No VAS-based outcomes were available for the two-implant group. For the four-implant group, ten VAS-based effect-size estimates were available, all originating from a single study population. Therefore, a pooled multi-level estimate could not be calculated for this group. For the ≥6-implant group, 20 effect-size estimates were included in the multi-level meta-analysis, resulting in a pooled Hedges’ *g* of 2.38 (95% CI: 1.79 − 2.97).

Multi-level meta-regression showed a significantly greater improvement in VAS-based outcomes for ≥6 implants compared with four implants (difference in Hedges’ *g* = 1.96; *p* < 0.001).

### Subgroup analyses

Subgroup analyses were performed according to implant number, type of restoration (fixed vs. removable), and follow-up period (12−24 vs.36−60 months). In all included studies, prostheses supported by two or four implants were removable, whereas both fixed and removable prostheses were represented among rehabilitations supported by six implants. Where the available data permitted, differences between subgroups were assessed using multi-level random-effects meta-regression.

#### Four vs. two implants: removable prosthesis and functional limitation

To further examine the limited evidence available for rehabilitation with two implants, the OHIP domain *functional limitation* was analyzed separately (see Table 7). At 12–24 months, no significant difference was observed between removable prostheses supported by two and four implants (difference in Hedges’ *g* = −0.10; 95% CI: −1.59−1.39; *p* = 0.893). This comparison was based on a multi-level meta-regression accounting for effect-size estimates originating from the same study.

At 36 − 60 months, the effect size was −0.19 (95% CI: −0.92, 0.55) for two implants and 1.25 (95% CI: 0.90–1.61) for four implants. Conventional random-effects meta-regression indicated a significant difference in favor of four implants (difference in Hedges’ *g* = 1.44; 95% CI: 0.63 − 2.25; *p* = 0.001). However, a multi-level meta-regression could not be performed for this comparison because of the limited amount of available data (see Table 7).

**Table 7:** Four vs. two implants, removable prosthesis, functional limitation (OHIP domain)

PROMS	n implants per patient	n effect size estimates	effect size [95%-CI]	p-value
OHIP 12–24 months	2 implants	2	1.38 [−1.06, 3.82]	
	4 implants	4	1.34 [0.93, 1.75]	
	4 vs 2 (RE*)		−0.10 [−1.59, 1.39]	0.893
OHIP 36–60 months	2 implants	1	−0.19 [−0.92, 0.55]	
	4 implants	3	1.25 [0.90, 1.61]	
	4 vs 2 (RE*)		1.44 [0.63, 2.25]	0.001

* RE: Differences between implant groups were assessed using random-effects meta-regression. A multi-level model was used for the 12–24-month comparison; at 36–60 months, a conventional random-effects meta-regression was performed because the available data did not permit multi-level analysis

#### Six vs. four implants: removable prostheses

To assess the association between implant number and patient-reported outcomes independently of prosthesis type, analyses were restricted to removable prostheses (Table 8). At 12 − 24 months, the pooled effect size was 1.13 (95% CI: 0.53 − 1.73; Fig. 2) for four implants and 1.86 (95% CI: 1.39 − 2.34; Fig. 5) for six implants. Multi-level meta-regression showed a significantly greater improvement with six implants (difference in Hedges’ g = 1.13; 95% CI: 0.53 − 2.06; *p* < 0.001).

**Table 8:** Six vs. four implants, removable prostheses

PROMS	n implants per patient	n effect size estimates	effect size [95%-CI]	p-value
OHIP total, VAS 12–24 months	4 implants	14	1.13 [0.53, 1.73]	
	6 implants	6	1.86 [1.39, 2.34]	
	6 vs 4 (RE*)		1.13 (0.53, 2.06)	<0.001
OHIP total, VAS 36–60 months	4 implants	6	1.11 [0.57, 1.65]	
	6 implants	12	2.15 [1.67–2.62]	
	6 vs 4 (RE*)		0.99 [0.05–1.94]	0.039

* RE: Random-effects meta-regression

At 36–60 months, the pooled effect sizes were 1.11 (95% CI: 0.57 − 1.65) for four implants and 2.15 (95% CI: 1.67 − 2.62) for six implants. The difference remained statistically significant in multi-level meta-regression (difference in Hedges’ g = 0.99; 95% CI: 0.05 − 1.94; *p* = 0.039).

#### Fixed vs. removable prostheses with six implants

To examine the potential influence of prosthesis type at a constant implant number, fixed and removable prostheses supported by six implants were compared at 12 − 24 months (Table 9). The pooled effect size was 2.59 (95% CI: 2.11 − 3.07) for fixed prostheses and 1.86 (95% CI: 1.39 − 2.34) for removable prostheses. Multi-level meta-regression showed no statistically significant difference between the two types of prostheses (difference in Hedges’ g = 0.65; 95% CI: −0.22−1.53; *p* = 0.143). No corresponding comparison was possible at 36–60 months because of insufficient data.

**Table 9:** Subgroup analysis comparing removable and fixed prostheses supported by six implants

PROMS	n implants per patient/type of prosthesis	n effect size estimates	effect size [95%-CI] (pooled Hedges‘g)	p-value
OHIP total, VAS	6 implants/removable	6	1.86 [1.39, 2.34]	
OHIP total, VAS	6 implants/fixed	17	2.59 [2.11, 3.07]	
	fixed vs. removable (RE*)		0.65 [−0.22, 1.53]	0.143

* RE: Random-effects meta-regression

### Publication bias

**Fig. 7:**
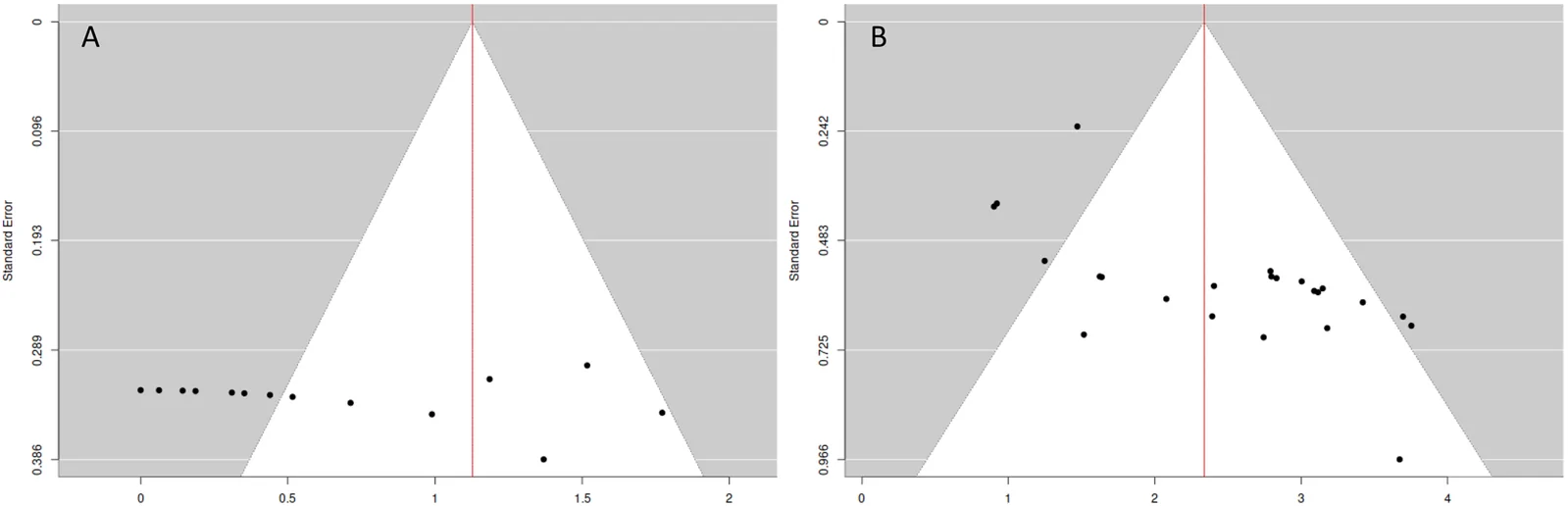
Funnel plots for the exploratory assessment of publication bias in the primary meta-analyses according to implant number. (**A**) Four-implant group; (**B**) ≥6-implant group. Effect sizes are expressed as Hedges’ g and plotted against their standard errors. The vertical red line indicates the pooled effect estimate

Funnel plots were generated for the main analyses and selected subgroup analyses for exploratory assessment of potential publication bias (Fig. 7 A/B). Visual inspection did not reveal a consistent pattern across analyses. However, interpretation was limited by the small number of independent studies and the inclusion of multiple correlated effect-size estimates derived from the same study populations. Therefore, no firm conclusions regarding funnel plot asymmetry or publication bias could be drawn.

## Discussion

This systematic review and meta-analysis evaluated longitudinal changes in patient-reported outcomes following implant-supported rehabilitation of the edentulous maxilla, with particular emphasis on implant number. Substantial improvements were observed across all implant groups, consistent with previous systematic reviews reporting favorable effects on OHRQoL and patient satisfaction following implant-based rehabilitation of the edentulous maxilla [18, 20, 24–26]. The largest pooled effect was observed for rehabilitation supported by six or more implants (Hedges’ g = 2.34; 95% CI: 1.85–2.83), with a significantly greater improvement than for four implants. Importantly, this difference remained significant when the analysis was restricted to removable prostheses, both at 12 − 24 months and at 36–60 months. Comparisons involving two implants remained inconclusive because of the limited evidence available. Sensitivity analyses further showed that the magnitude of the association varied according to PROM type, being more pronounced for VAS-based than for OHIP-based outcomes.

To our knowledge, this is the first meta-analysis to quantitatively compare longitudinal changes in patient-reported outcomes according to implant number specifically in the edentulous maxilla. Previous systematic reviews have addressed related questions but differed in scope and methodology [16–18, 20, 24–26]. Of particular relevance, Abou-Ayash et al. used standardized pre- to post-treatment effect sizes (Hedges’ g) to synthesize patient-reported outcomes assessed using different instruments, including OHIP- and VAS-based measures [24]. The methodological approach of the present analysis was informed by this work. However, their analysis predominantly included mandibular rehabilitation, with only limited evidence from maxillary cohorts. The present analysis extends this approach by focusing exclusively on the edentulous maxilla, comparing outcomes according to implant number, and accounting for dependencies among multiple effect-size estimates from the same study population using a multi-level model. This maxilla-specific approach is particularly relevant because baseline impairment and the magnitude of treatment-related improvement may differ substantially between the two arches.

The greater improvement observed with ≥6 compared with four implants adds to previous evidence in which an effect of implant number on patient-reported outcomes has remained uncertain. Di Francesco et al. reported high patient satisfaction with maxillary overdentures supported by both four and six splinted implants without demonstrating a significant difference between the two concepts [26]. Similarly, recent evidence on fixed full-arch rehabilitation has not established a consistent association between implant number and PROMs [17]. The present analysis differs by quantifying standardized longitudinal change from the pre-treatment condition. Accordingly, the observed difference reflects the magnitude of improvement rather than absolute post-treatment OHRQoL or satisfaction.

Prosthetic design represents an important consideration when interpreting this finding because all four-implant restorations included in the present meta-analysis were removable, whereas the ≥6-implant group comprised both fixed and removable restorations. However, the association with implant number persisted when the analysis was restricted to removable prostheses. Six-implant removable restorations showed a significantly greater improvement than four-implant restorations at 12 − 24 months (difference in Hedges’ *g* = 1.13; 95% CI: 0.53–2.06; *p* < 0.001), and this difference remained significant at 36–60 months (difference in Hedges’ *g* = 0.99; 95% CI: 0.05–1.94; *p* = 0.039). Conversely, no statistically significant difference was observed between fixed and removable prostheses supported by six implants at 12–24 months (*p* = 0.143). This is broadly consistent with Yao et al., who reported substantial patient-reported benefits with both prosthetic concepts [25]. Together, these findings strengthen the observation that the difference between four and ≥6 implants cannot be explained solely by the inclusion of fixed restorations in the ≥6-implant group. At the same time, the absence of a statistically significant overall difference between fixed and removable prostheses should not be interpreted as evidence of equivalence.

Other prosthetic characteristics may nevertheless contribute to patient perception. Fixed and removable restorations differ in aspects such as speech, esthetics, hygiene and maintenance, while attachment systems may influence retention and handling. Notably, all removable prostheses included in the present analysis were designed without palatal coverage, making palatal extension an unlikely explanation for the difference observed between four- and six-implant removable concepts. The available evidence did not permit further robust stratification according to attachment system or individual functional domains.

Evidence for two-implant rehabilitation of the edentulous maxilla remains limited. Only one study contributed an overall OHIP score to the primary analysis, precluding a pooled multi-level estimate. No significant difference between two and four implants was observed for OHIP functional limitation at 12 months, whereas the exploratory 36–60-month analysis favored four implants. These PROM findings should be considered together with evidence on clinical predictability. Previous meta-analytic evidence demonstrated higher implant loss rates for maxillary overdentures supported by fewer than four implants, and the recent Global Consensus recommends at least four appropriately distributed implants for maxillary implant overdentures to reduce implant failure [14, 41].

Although two-implant maxillary overdentures can provide substantial patient-reported benefits, a prospective pilot study by our group reported an implant survival rate of 68% after three years with two narrow-diameter titanium-zirconium implants in the maxilla [37, 40, 42]. Thus, favorable PROMs with two implants need to be considered alongside the more limited evidence for clinical predictability.

The sensitivity analyses according to PROM type provide further insight into the primary findings. OHIP- and VAS-based analyses showed the same direction of effect, but the difference between implant groups was more pronounced for VAS-based outcomes. When restricted to OHIP, improvements were observed across all implant groups and were numerically greater with ≥6 than with four implants, but the between-group difference was not statistically significant. This discrepancy is plausible because OHIP and VAS-based assessments capture different aspects of patient experience. The OHIP is a validated multidimensional measure of OHRQoL, whereas VAS assessments commonly address specific aspects such as satisfaction, retention, mastication, esthetics, speech, or comfort [4, 6, 7]. Differences between treatment concepts may therefore be more readily reflected in specific VAS domains than in a global OHRQoL score.

The VAS findings nevertheless require cautious interpretation because all ten VAS-based effect-size estimates in the four-implant group originated from a single study population. Although the multi-level model accounts for dependencies among multiple estimates from the same study, these estimates do not represent independent replication across cohorts. Moreover, individual VAS items are not psychometrically equivalent to validated multi-item OHRQoL instruments. Greater standardization of PROM selection and reporting would facilitate future comparisons between treatment concepts [4, 12, 22].

From a clinical perspective, the present findings indicate that implant number is associated with the magnitude of patient-reported improvement following rehabilitation of the edentulous maxilla, with the largest improvements observed for ≥6 implants. The persistence of the difference between four and six implants within removable treatment concepts supports the relevance of implant number beyond prosthetic design alone. Nevertheless, treatment selection also incorporates anatomical conditions, prosthetic requirements, anticipated maintenance, treatment burden, costs, and patient preferences. Recent evidence showing favorable survival but substantial maintenance requirements for implant-assisted maxillary restorations further emphasizes the importance of considering patient-reported and clinical outcomes together [43].

### Strengths and limitations

A major strength of the present review is its restriction to the edentulous maxilla, thereby avoiding potential confounding from mandibular treatment effects. Longitudinal changes were standardized using Hedges’ g, allowing patient-reported outcomes assessed using different scales to be synthesized on a common metric. The use of a multi-level random-effects model further allowed multiple effect-size estimates from the same study population to be retained while accounting for their statistical dependence and reducing the risk of pseudo-replication and artificial precision. Sensitivity analyses according to PROM type and subgroup analyses according to prosthetic design and follow-up provided additional insight into the robustness and limitations of the primary findings.

Several limitations should nevertheless be considered. The number of independent studies was limited, particularly for two-implant rehabilitation, and several pooled estimates were based on multiple outcomes originating from relatively few study populations. Implant number and prosthetic design could not be fully separated because no eligible study provided the required longitudinal PROM data for fixed full-arch rehabilitation supported by four implants. Heterogeneity in PROM instruments, outcome domains, attachment systems, implant distribution, and treatment protocols further limits direct comparability between studies. Analyses were based on aggregate data, precluding adjustment for potentially relevant patient-level characteristics and baseline differences. This is particularly relevant when interpreting standardized changes from baseline, as the magnitude of improvement may depend partly on the level of impairment before treatment. The VAS sensitivity analysis requires particular caution because all four-implant VAS estimates originated from a single study population.

## Conclusions

Implant-supported rehabilitation of the edentulous maxilla was associated with substantial improvements in patient-reported outcomes across all implant numbers. The largest improvements were observed with ≥6 implants, with a significant advantage over four implants that persisted when the analysis was restricted to removable prostheses. Although the magnitude of this association varied according to PROM type and evidence for two-implant rehabilitation remains limited, the findings support implant number as a relevant factor in patient-reported treatment benefit.

## Electronic supplementary material

Below is the link to the electronic supplementary material.

Supplementary Material 1

Supplementary Material 2

## Data Availability

The data that supports the findings of this study are available on request from the corresponding author.

## Abbreviations

CI: Confidence interval
dPROM: dental patient-reported outcome measure
MINORS: Methodological index for non-randomized studies
OHIP: Oral health impact profile
OHRQoL: Oral health-related quality of life
PRISMA: Preferred reporting items for systematic reviews and meta-analyses
PROM: Patient-reported outcome measure
RCT: Randomized controlled trial
REML: Restricted maximum likelihood
RoB 2: Revised Cochrane risk of bias tool
ROBINS-I: Risk of bias in non-randomized studies of interventions
VAS: Visual analogue scale

## Declarations

### PROSPERO registration

This systematic review was registered on https://www.crd.york.ac.uk/prospero/ on 05 June 2025 with the registration number CRD420251055557. Start date of systematic review: May 18th 2025.

### AI statement

During the preparation of this work the authors used Chat GPT and DeepL in order to improve readability and language. After using these tools, the authors reviewed and edited the content as needed and take full responsibility for the content of the publication.
